# Central Nervous System Cryptococcosis in Patients With Sarcoidosis: Comparison With Non-sarcoidosis Patients and Review of Potential Pathophysiological Mechanisms

**DOI:** 10.3389/fmed.2022.836886

**Published:** 2022-03-29

**Authors:** Renaud Prevel, Vivien Guillotin, Sébastien Imbert, Patrick Blanco, Laurence Delhaes, Pierre Duffau

**Affiliations:** ^1^CHU Bordeaux, Internal Medicine Department, Bordeaux, France; ^2^Univ Bordeaux, Centre de Recherche Cardio-Thoracique de Bordeaux, Inserm UMR 1045, Bordeaux, France; ^3^CHU Bordeaux, Mycology-Parasitology Department, CIC 1401, Bordeaux, France; ^4^CHU Bordeaux, Immunology Department, Bordeaux, France; ^5^Univ Bordeaux, CNRS ImmunoConcEpT UMR 5164, Bordeaux, France

**Keywords:** cryptococcal meningitis, sarcoidosis, innate immunity, humoral immune response, anti-GM-CSF autoantibodies

## Abstract

**Introduction:**

*Cryptococcus* spp. infection of the central nervous system (CINS) is a devastating opportunistic infection that was historically described in patients with acquired immunodeficiency syndrome (AIDS). *Cryptococcus* spp. infections are also associated with sarcoidosis; the impairment of cell-mediated immunity and long-term corticosteroid therapy being evoked to explain this association. Nevertheless, this assertion is debated and the underlying pathophysiological mechanisms are still unknown. The aims of this study were (i) to describe the clinical and biological presentation, treatments, and outcomes of CINS patients with and without sarcoidosis and (ii) to review the pathophysiological evidence underlying this clinical association.

**Patients and Methods:**

Every patient with positive cerebrospinal fluid (CSF) cryptococcal antigen testing, India ink preparation, and/or culture from January 2015 to December 2020 at a tertiary university hospital were included, and patients with sarcoidosis were compared with non-sarcoidosis patients. Quantitative variables are presented as mean ± SD and are compared using the Mann-Whitney Wilcoxon rank-sum test. Categorical variables are expressed as the number of patients (percentage) and compared using the χ^2^ or Fisher's tests.

**Results:**

During the study period, 16 patients experienced CINS, of whom 5 (31%) were associated with sarcoidosis. CINS symptoms, biological, and CSF features were similar between CINS patients with and without sarcoidosis except regarding CD4 cells percentages and CD4/CD8 ratio that was higher in those with sarcoidosis (47 ± 12 vs. 22 ± 18, *p* = 0.02 and 2.24 ± 1.42 vs. 0.83 ± 1.10, *p* = 0.03, respectively). CINS patients with sarcoidosis had less often positive blood antigen testing than those without sarcoidosis (2/5 vs. 11/11, *p* = 0.02). CINS patients with and without sarcoidosis were treated with similar drugs, but patients with sarcoidosis had a shorter length of treatment. CD4 cell levels do not seem to explain the association between sarcoidosis and cryptococcosis.

**Conclusion:**

Sarcoidosis was the most frequently associated condition with CINS in this study. CINS patients associated with sarcoidosis had overall similar clinical and biological presentation than CINS patients associated with other conditions but exhibited a lower rate of positive blood cryptococcal antigen testing and higher CD4/CD8 T cells ratio. Pathophysiological mechanisms underlying this association remain poorly understood but B-1 cell deficiency or lack of IgM could be a part of the explanation. Another plausible mechanism is the presence of anti-granulocyte-macrophage colony-stimulating factor (GM-CSF) antibodies in a subset of patients with sarcoidosis, which could impair macrophage phagocytic function. Further studies are strongly needed to better understand those mechanisms and to identify at-risk patients.

## Introduction

Sarcoidosis is a systemic disease characterized by the formation of non-caseating epithelioid granulomas in several organs, mainly the lungs and the lymphatic system (1). It affects between 4.7 and 64 per 100,000 people and its prevalence varies from 1.0 to 64 per 100,000 per year (2). It can occur at any age of life, but with a particular proclivity for young adults. Spontaneous resolution occurs in about 60% of patients. Nevertheless, chronic and progressive forms are not rare and about 20% of patients have permanent clinical symptoms due to fibrotic lesions in the involved organs (2). Mortality is thought to be up to 5–10% (1), mainly associated with severe pulmonary fibrosis or, less usually, with cardiac or central nervous system involvement. Even if its exact origin remains unknown, the persistence of an unidentified antigen in individuals with genetic predisposition is supposed to trigger a pro-inflammatory Th1 response, leading to the formation of these granulomas. Sarcoidosis is characterized by a paradoxical immune status, i.e., an exaggerated immune response within granulomas, in contrast to various immune defects as stated by anergy to tuberculin test and the occurrence of opportunistic infections (3, 4), with corticosteroid therapy being a constant risk factor (4).

The first suspected opportunistic infections were mycobacterial ones but, except in patients receiving anti-TNFα treatments, sarcoidosis does not seem to be at particular risk for tuberculosis nor for any other mycobacterial infections. In fact, tuberculosis is less frequent, more typical, and not associated with immune reconstitution inflammatory syndrome in sarcoidosis patients compared with patients with acquired immunodeficiency syndrome (AIDS) (5). *Mycobacterium avium* complex infections were organ-specific in sarcoidosis patients and not disseminated as in patients with AIDS with no correlation with CD4 count (6). On the contrary, opportunistic infections such as progressive multifocal leukoencephalopathy (PML) and aspergillosis have been described in patients with sarcoidosis (4). Sarcoidosis is an underlying disease in 8–9% of PML cases with most patients who had not received any immunosuppressive drug (7, 8). Moreover, sarcoidosis represents a risk factor in 7–17% of chronic pulmonary aspergillosis (9, 10), and chronic pulmonary aspergillosis complicates around 2% of sarcoidosis (11, 12) with long-term corticosteroid therapy being a major risk factor for invasive aspergillosis, increasing in parallel with treatment dose and duration. Nocardiosis, histoplasmosis, and pneumocystosis are also reported in patients with sarcoidosis, but less frequently when compared with patients with rheumatological disorders who have more profound immunosuppression, especially after corticosteroid exposure (4).

*Cryptococcus* spp. infection of the central nervous system (CINS) is a devastating opportunistic infection that was first described in patients affected by AIDS historically representing about 70% of cryptococcal infections (13, 14). *Cryptococcus* spp. is responsible for one million infections per year among human immunodeficiency virus (HIV)-infected patients in the world; of these, approximately 625,000 die (15). *Cryptococcus neoformans* and *Cryptococcus gattii* are the most common and major pathogenic species complex in the genus *Cryptococcus* (16). *C. gattii* shares major virulence determinants with *C. neoformans* and was previously thought to be a subtype of *C. neoformans*, but genomic and transcriptomic studies revealed distinctions leading to recognize *C. gattii* as a unique species (17). Both species usually cause pulmonary or central nervous system (CNS) infections, but they differ in epidemiology, clinical features, and pathophysiology (18). *C. gattii* has traditionally been considered as a “tropical or subtropical fungus” despite the fact that, even before the North American outbreak, a large proportion of disease in Australia occurred in its southern temperate region. It has now been isolated from human and animal samples worldwide (17). Shifts in the appreciation of the clinical epidemiology of *C. gattii* in the past two decades include the recognition that it affects hosts known to be immunocompromised (including those with HIV/AIDS) as well as hosts presumed to be immunocompetent (17). The patients affected by other immunocompromising conditions such as sarcoidosis or others (lymphoproliferative disorders, malignancy diseases, organ transplant, and/or immunosuppressive therapy) are so predisposed to CINS, possibly caused by both *C. neoformans* and *C. gattii* (19–21) and cryptococcal infections affect 0.8 per 100,000 HIV-negative inhabitants (22). Their proportion is increasing with the advances in the care of HIV-infected patients (23, 24) and the increasing number of patients receiving immunosuppressive drugs. T cell-mediated immunity is the major pathway of defense against *Cryptococcus* spp. with a key role for Th1-Th2 imbalance resulting in impaired TNFα, IL-12, and IFN-γ production (25), and patients with AIDS are known to have impaired T cell-mediated immunity. The use of corticosteroids is a well-recognized risk factor for cryptococcosis (20, 22, 26), as for other opportunistic infections, with an estimated risk of fungal infections 1.5 times greater (95% CI: 1.3–1.9) in patients taking corticosteroids compared with naïve controls (11). The risk is especially increased for dosages exceeding 20 mg/day (27). In fact, corticosteroids cause dysregulation of Th1/Th2 T helper cells to balance favoring Th2 cytokines response and decrease cooperation with B cells (28). They are also responsible for a reduction in monocyte-macrophage functions by reducing chemotaxis, phagocytosis, and production of IL-1, IL-6, and TNF-α (29). The impairment of cell-mediated immunity (low CD4 cell count and lower CD4/CD8 ratio) has also been suggested as a risk factor for opportunistic infections in patients with sarcoidosis (11, 30, 31). Inconsistent with this hypothesis, numerous patients with sarcoidosis suffering from CINS reported in the literature were not receiving long-term corticosteroids therapy (11, 32, 33). A case-control study comparing sarcoidosis patients with and without CINS further confirmed corticosteroids therapy as a risk factor for CINS (34) but no association between the risk of opportunistic infection and severe CD4 lymphocytopenia was found.

To the best of our knowledge, no case-control study compared CINS patients with and without sarcoidosis to further address the underlying mechanisms. The aims of this study were to describe the clinical and biological presentation, treatments, and outcomes of CINS patients with and without sarcoidosis and to review the pathophysiological evidence underlying this clinical association.

## Patients and Methods

### Study Design and Inclusion Criteria

This study was conducted at Bordeaux University Hospital including every patient with positive cerebrospinal fluid (CSF) cryptococcal antigen testing, India ink preparation, and/or culture from January 2015 to December 2020.

Cerebrospinal fluid cryptococcal antigen testing was performed using CryptoPS test (BIOSYNEX®) and blood cryptococcal antigen testing using CALAS® (Meridian Bioscience). CSF samples were processed with India ink preparation and incubated on Sabouraud Agar + Chloramphenicol + Gentamicin media (Bio-Rad). Blood samples were incubated on BACT/ALERT® FA (bioMérieux) culture bottles. Identification of growing isolates was performed using MALDI-TOF mass spectrometry (Microflex®, Bruker Daltonics).

Diagnosis of sarcoidosis was retrospectively confirmed according to the current recommendations (35): (i) clinical and paraclinical features consistent with sarcoidosis, (ii) an histopathological analysis revealing non-caseating granuloma except for patients presenting Löfgren's syndrome, and (iii) exclusion of other possible etiologies, including other granulomatous disorders. Data were retrospectively collected from the electronic medical records, and the electronic worksheet was completed by two medical intensive care residents.

### Statistical Analyses

No statistical sample size calculation was performed *a priori*, and the sample size was equal to the number of patients with positive CSF cryptococcal antigen testing and/or culture during the study period. Quantitative variables are presented as mean ± SD and compared using the Mann-Whitney Wilcoxon rank-sum test. Categorical variables are expressed as the number of patients (percentage) and compared using the χ^2^ or Fisher's tests. All statistical tests were 2-tailed, and statistical significance was defined as *p* < 0.05. Statistical analyses were assessed using the R version 3.6.0 statistical software (R Foundation for Statistical Computing Vienna, Austria).

### Ethics

According to the French law and the French Data Protection Authority, the handling of these data for research purposes was declared to the Data Protection Officer of the Bordeaux University Hospital. Patients (or their relatives, if any) were notified about the anonymized use of their healthcare data *via* the department's booklet.

## Results

### High Proportion of Patients With Sarcoidosis in Patients With CINS

During the study period, 16 patients presented CINS, of whom five were associated with sarcoidosis, four with an onco-hematological disease including two patients treated with ibrutinib, three patients with AIDS, two with kidney transplantation, one with autoimmune hepatitis, and one with epilepsy associated to Arnold-Chiari malformation (Table 1).

**Table 1:** Characteristics of patients diagnosed with *Cryptococcus* sp. infection of the central nervous system.

	**Sarcoidosis** **(*n* = 5)**	**Non-sarcoidosis** **(*n* = 11)**	***p*-value**
Age	49 ± 11.7	55 ± 18.6	0.53
Male	4 (80%)	8 (78%)	1.00
Etiology of immunosuppressive state in non-sarcoidosis patients			
Onco-hematology	-	4 (39%)	-
HIV	-	3 (30%)	-
Kidney transplant	-	2 (20%)	-
Auto-immune hepatitis	-	1 (10%)	-
Epilepsy with Arnold-Chiari malformation	-	1 (10%)	-
Characteristics of sarcoidosis			
Known sarcoidosis	5	-	-
Duration of sarcoidosis evolution (months)	36 ± 31	-	-
Lung involvement	3 (60%)	-	-
Mediastinal adenopathy	2 (40%)	-	-
Central nervous system	1 (20%)	-	-
Peripheral nervous system	1 (20%)	-	-
Muscular involvement	1 (20%)	-	-
Liver involvement	1 (20%)	-	-
Joint, eye, skin, kidney, parotid	0 (0%)	-	-
Calcemia (mmol/L) Normal values: 2.2–2.6 mmol/L	2.32 ± 0.14	-	-
Angiotensin-converting enzyme Normal values: 20–80 IU/L	39 ± 27	-	-
Immunosuppressive drugs			
Past therapy with corticosteroids	4 (80%)	6 (59%)	0.59
Past other immunosuppressive drug	3 (60%)	5 (49%)	1.00
Cyclophosphamide	-	3 (30%)	-
CHOEP	-	1 (10%)	-
RCD then R-bendamustine	-	1 (10%)	-
Azathioprine	1 (20%)	1 (10%)	-
Mycophenolate-mofetil	1 (20%)	0 (0%)	-
Methotrexate	1 (20%)	0 (0%)	-
Current therapy with corticosteroids	4 (80%)	5 (49%)	0.31
Current other immunosuppressive drug	1 (20%)	5 (49%)	0.59
Ibrutinib	-	2 (20%)	-
Tacrolimus	-	2 (20%)	-
Mycophenolate-mofetil	0 (0%)	1 (10%)	-
Azathioprine	1 (20%)	2 (20%)	-

*CHOEP, cyclophosphamide, doxorubicin, vincristine, etoposide and prednisone; HIV, human immunodeficiency virus; IU, international unit; R, rituximab; R-CD, rituximab–cyclophosphamide-dexamethasone*.

The median duration of sarcoidosis at cryptococcosis diagnosis was 36 ± 31 months. Patients with sarcoidosis were mostly men (4/5) with a median age of 49 ± 11.7 years, of whom three had lung involvement, two with mediastinal adenopathy, one with central and peripheral nervous system involvement, and one with muscular involvement. One of them only had liver involvement. Of these five patients, four were receiving corticosteroids therapy at the time of cryptococcosis diagnosis and three of them had previously received another immunosuppressive drug (one with azathioprine, one with mycophenolate-mofetil, and one with methotrexate) (Table 1). In patients receiving immunosuppressive drugs, only azathioprine was still ongoing at the time of cryptococcosis onset. No other previous opportunistic infection was reported among those patients with sarcoidosis.

In non-sarcoidosis patients, 6/11 previously received corticosteroid therapy, still ongoing for five of them, and 5/11 previously received other immunosuppressive drugs: 3 cyclophosphamide, 1 CHOEP, 1 R-CD then R-bendamustine, and 1 azathioprine. At the time of diagnosis, five of them were receiving immunosuppressive drugs: 2 ibrutinib, 2 tacrolimus, 2 azathioprine, and 1 mycophenolate-mofetil.

### CINS Patients With Sarcoidosis Have a Similar Clinical and Biological Presentation at Diagnosis Than Those Without Sarcoidosis but Lower Positive Blood Cryptococcal Antigen Testing

All of the five patients with sarcoidosis reported fever and headaches as symptoms of CINS. Two of them exhibited neck stiffness, two seizures, one behavior abnormalities, and one focal neurological deficit, being similar for all those features to non-sarcoidosis patients (Table 2). CSF cell count, protein, lactate, and glucose levels as a proportion of positive CSF antigen testing or culture were comparable between CINS patients with and without sarcoidosis. Each included patient had blood antigen testing that was less often positive (2/5 vs. 11/11, *p* = 0.02) in patients with sarcoidosis than in those without sarcoidosis (Table 2). Each cultivated strain but one was from *C. neoformans* species. Microbiological results on a patient basis are provided (Table 3).

**Table 2:** Patients' presentation and immunological assessment at diagnosis of *Cryptococcus* spp. infection of central nervous system.

	**Sarcoidosis (*n* = 5)**	**Non-sarcoidosis (*n* = 11)**	***p*-value**
**Patients' presentation**			
Fever	5 (100%)	9	1.00
Headaches	5 (100%)	9	1.00
Neck stiffness	2 (40%)	3	1.00
Seizures	2 (40%)	1	0.21
Dizziness	1 (20%)	2	1.00
Behavior abnormalities	1 (20%)	2	1.00
Focal neurologic deficit	1 (20%)	1	1.00
Confusion	0 (0%)	4	0.24
Asthenia	4 (80%)	8	1.00
Weight loss	2 (40%)	7	0.33
Neutrophils count (/mm^3^)	8,070 ± 4,585	6,762 ± 4,584	0.65
Platelets count (/mm^3^)	297,600 ± 154,063	282,909 ± 194,531	0.65
Lymphocytes count (/mm^3^)	858 ± 567	797 ± 667	0.82
Positive blood culture	0	0	1.00
**Positive blood antigen**	**2**	**11**	**0.02**
CSF cells count (/mm^3^)	143 ± 112	544 ± 1,087	0.57
%age of lymphocytes in CSF	42.8 ± 26.1	63.2 ± 39.6	0.30
CSF protein (/mm^3^)	1.82 ± 1.61	1.06 ± 0.71	0.31
CSF lactates (mmol/L)	5.7 ± 3.8	7.2 ± 12	0.42
CSF glucose level (mmol/L)	2.37 ± 0.46	3.13 ± 1.59	0.21
Positive Ink coloration	2	5	1.00
Positive CSF culture	2	8	0.30
Positive CSF antigen	5	11	1.00
Meningitis on MRI	2/4	0/7	0.11
Encephalitis on MRI	2/4	2/7	0.58
Time from onset of symptoms to diagnosis (days)	65 ± 68	34 ± 30	0.25
Pitfalls in diagnosis	3	4	0.60
	1 neuro-sarcoidosis 1 viral meningitis 1 pneumonitis	2 sinusitis 1 bronchitis 1 pneumonitis	-
Concomitant pulmonary involvement	1	4	1.00
**Immunological assessment**			
CD4 lymphocytes count (/mm^3^)	424 ± 242	242 ± 340 w/o AIDS patients: 355 ± 374	0.11 0.43
**CD4 lymphocytes proportion (%)**	**47** **±12**	**22** **±18** **w/o AIDS patients:** **29** **±8.8**	**0.02 0.03**
CD8 lymphocytes count (/mm^3^)	202 ± 118	356 ± 191 w/o AIDS patients: 314 ± 198	0.15 0.33
**CD8 lymphocytes proportion (%)**	**20** **±4.5**	**47** **±21** **w/o AIDS patients:** **34** **±12**	**0.02 0.01**
**CD4/CD8 lymphocytes ratio**	**2.24** **±1.42**	**0.83** **±1.10** w/o AIDS patients: 0.96 ± 0.69	**0.03** 0.08
B lymphocytes count (/mm^3^)	92 ± 99	157 ± 290	0.79
B lymphocytes proportion (%)	13.8 ± 9.91	6.99 ± 7.72	0.25
Gamma globulins level (g/L)	6.57 ± 1.53	11.46 ± 8.11	0.48
IgG level (g/L)	8.45 ± 4.88	8.49 ± 5.09	0.89
IgA level (g/L)	0.99 ± 0.02	2.12 ± 1.80	0.67
IgM level (g/L)	1.18 ± 0.25	0.85 ± 0.59	0.50

*CSF, cerebrospinal fluid; MRI, magnetic resonance imaging. Bold values mean statistically significant*.

**Table 3:** Microbiologic results on a patient basis.

	**Sex**	**Age** **(years old)**	**Associated condition**	**Blood culture**	**Blood antigen testing**	**Titer (dilution)**	**India ink**	**CSF culture**	**Isolated specie**	**CSF** **antigen testing**	**Titer (dilution)**
Patient 1	M	48	Sarcoidosis	Negative	Negative	-	Positive	Negative	-	Positive	1/128
Patient 2	M	40	Sarcoidosis	Negative	Negative	-	Negative	Positive	*C. neoformans*	Positive	NA
Patient 3	F	35	Sarcoidosis	Negative	Negative	-	Negative	Negative	-	Positive	NA
Patient 4	M	59	Sarcoidosis	Negative	Positive	1/512	Negative	Negative	-	Positive	1/16
Patient 5	M	62	Sarcoidosis	Negative	Positive	1/256	Positive	Positive	*C. neoformans*	Positive	1/512
Patient 6	M	85	Hematological malignancy	Negative	Positive	1/128	Negative	Positive	*C. neoformans*	Positive	1/1,024
Patient 7	M	65	Hematological malignancy	Negative	Positive	1/1,024	Positive	Positive	*C. neoformans*	Positive	1/1,024
Patient 8	F	55	Hematological malignancy	Negative	Positive	1/12	Negative	Negative	-	Positive	1/8
Patient 9	M	76	Hematological malignancy	Negative	Positive	1/128	Negative	Negative	-	Positive	1/16
Patient 10	F	40	HIV	Negative	Positive	1/64	Positive	Positive	*C. neoformans*	Positive	1/256
Patient 11	M	44	HIV	Negative	Positive	1/8,192	Positive	Positive	*C. neoformans*	Positive	1/4,096
Patient 12	M	32	HIV	Negative	Positive	1/2,048	Negative	Positive	*C. neoformans*	Positive	1/2,048
Patient 13	M	69	Kidney transplantation	Negative	Positive	1/256	Positive	Positive	-	Positive	1/512
Patient 14	M	46	Kidney transplantation	Negative	Positive	1/128	Negative	Negative	-	Positive	1/1,024
Patient 15	M	63	Auto-immune hepatitis	Negative	Positive	1/2	Negative	Positive	*C. neoformans*	Positive	1/32
Patient 16	F	27	Epilepsy associated to Arnold-Chiari malformation	Negative	Positive	1/1,024	Positive	Positive	*C. gattii*	Positive	1/2,048

*CSF, cerebrospinal fluid; HIV, human-immunodeficiency virus; F, female; M, male; NA, not available*.

### CINS Patients With Sarcoidosis Exhibit a Higher CD4/CD8 T Cells Ratio Than Those Without Sarcoidosis

The blood B lymphocytes count and proportion and the blood gamma globulin levels were similar between CINS patients with and without sarcoidosis (Table 2).

Regarding cellular immunity, CD4 cells proportion among total lymphocyte blood count and CD4/CD8 ratio were higher in CINS patients with sarcoidosis than in those without sarcoidosis (47 ± 12 vs. 22 ± 18, *p* = 0.02 and 2.24 ± 1.42 vs. 0.83 ± 1.10, *p* = 0.03, respectively) (Table 2). The differences in CD4 and CD8 T cells proportion remained statistically significant after excluding patients with AIDS from non-sarcoidosis patients (Table 2).

### CINS Patients With Sarcoidosis Have a Shorter Duration of Antifungal Treatment but Similar Outcomes Than Those Without Sarcoidosis

As stated in current therapeutic guidelines, patients with CINS mostly received a 2-week association of *intravenous* liposomal B amphotericin (3 mg/kg per day) and flucytosine (25 mg/kg every 6 h) as initial therapy (4/5 in patients with sarcoidosis vs. 8/11 in patients without sarcoidosis, *p* = 1.00) and then fluconazole *per os* (400 mg per day for 8 weeks then 200 mg per day) as maintenance therapy (4/5 in patients with sarcoidosis vs. 10/11 in patients without, *p* = 1.00) (Table 4). Nevertheless, patients with sarcoidosis were treated for a shorter duration (34 ± 29 days vs. 66 ± 13, *p* = 0.04) than those without sarcoidosis with a similar death rate and long-term sequelae (*p* = 1.00 for both) (Table 4).

**Table 4:** Management and outcomes of *Cryptococcus* spp. infection of the central nervous system.

	**Sarcoidosis (*n* = 5)**	**Non-sarcoidosis** **(*n* = 11)**	***p*-value**
Time from diagnosis to treatment initiation (days)	0.20 ± 0.45	0.45 ± 0.93	0.76
Initial 2-week liposomal B amphotericin and flucytosine bitherapy^*^	4	8	1.00
Fluconazole maintenance therapy^**^	4	10	1.00
**Treatment total duration (days)**	**34** **±29**	**66** **±13**	**0.04**
Death due to *Cryptococcosis*	1	1	1.00
Long-term sequelae	1/4 Cerebellar ataxia	2/10 Motor function	1.00 -

* *Intravenous liposomal B amphotericin: 3 mg/kg per day. Intravenous flucytosine: 25 mg/kg every 6 h*.

** *Fluconazole per os: 400 mg per day for 2 weeks, then 200 mg per day. Bold values are statistically significant*.

## Discussion

In this study, sarcoidosis is the most prevalent disease associated with CINS: 5 CINS over a period of 6 years with sarcoidosis accounting for 31% of all CINS and 38% (5/13) of the HIV-negative patients. CINS patients with sarcoidosis have a similar clinical and biological presentation at diagnosis but exhibit a higher CD4/CD8 T cells ratio than those without sarcoidosis. Importantly, CINS patients with sarcoidosis exhibited a lower rate of positive blood antigen testing compared with those without sarcoidosis. In front of CINS suspicion in a patient with sarcoidosis, blood cryptococcal antigen testing should be reiterated if negative when a reasonable clinical probability is assessed. CSF antigen testing should also be performed in case of clinical suspicion even if blood antigen testing is negative. CINS patients with sarcoidosis had a shorter duration of treatment than those without sarcoidosis. Every patient was treated based on specialized team stewardship. Shorter duration could be explained by the absence of severe quantitative CD4 count defect, the rarer use of immunosuppressive drugs, and the lower proportion of blood positive antigen testing in patients with sarcoidosis than in other patients. Recommendations regarding the duration of treatment for CINS have been established in AIDS and solid-organ transplant patients, and to the best of our knowledge, no consensus exists in patients with sarcoidosis.

Contrary to our findings, only very few cases were identified in the main case-control study (cryptOsarc study) comparing sarcoidosis patients with and without cryptococcosis (34). This study included 18 sarcoidosis patients with cryptococcosis, of whom 13 had CINS, over a period of 25 years. These patients were compared with 36 sarcoidosis patients without cryptococcosis. Sarcoidosis accounted for 0.6% of all cryptococcosis patients and 2.9% of the HIV-negative cryptococcosis patients. This discrepancy could be explained by the major therapeutic advances in the care of HIV-infected patients and by increasing the awareness of practitioners regarding the risk of cryptococcosis in other immunosuppressive conditions leading to more frequent testing. Among these 18 sarcoidosis patients with cryptococcosis included in the cryptOsarc study, 4 (23%) had sarcoidosis diagnosed at the time of infection and the 14 others had a median duration of 1,005 days (0–5,876) from the onset of the sarcoidosis. Notably, 12 out of the 18 patients (67%) had previously been treated with corticosteroids with a median therapy duration of 137 days (0–5,695), and a median dose of 18 mg/day (0–55) and two of them were receiving immunosuppressive drugs (cyclophosphamide for one and methotrexate plus infliximab for the other). The median level of CD4 lymphocytes at the time of cryptococcosis diagnosis was 145/mm^3^ (55–1,300). Compared with those free from cryptococcosis, patients with sarcoidosis and cryptococcosis were mostly men (72 vs. 47%, *p* = 0.145), they were younger (median age 28 vs. 42, *p* = 0.0004) and extra-pulmonary involvement was more frequent (83 vs. 56%, *p* = 0.069), including cardiac involvement, neurosarcoidosis or naso-sinusal, and/or parotid involvement. Factors associated with cryptococcosis in those patients with sarcoidosis were extra-thoracic sarcoidosis (*p* = 0.055) and possibly the intake of corticosteroid therapy or not (*p* = 0.123). Nevertheless, in this cryptOsarc study, only two-thirds of these patients received corticosteroids therapy and none of them experienced other opportunistic infections suggesting a specific susceptibility. In fact, patients with sarcoidosis are known to exhibit a decreased T cell response to cryptococcal antigen *in vitro* (36). As the pathophysiology of sarcoidosis still remains poorly understood, an unexplained primary immunodeficiency could favor both the occurrence of sarcoidosis and cryptococcosis (36).

**Figure 1:**
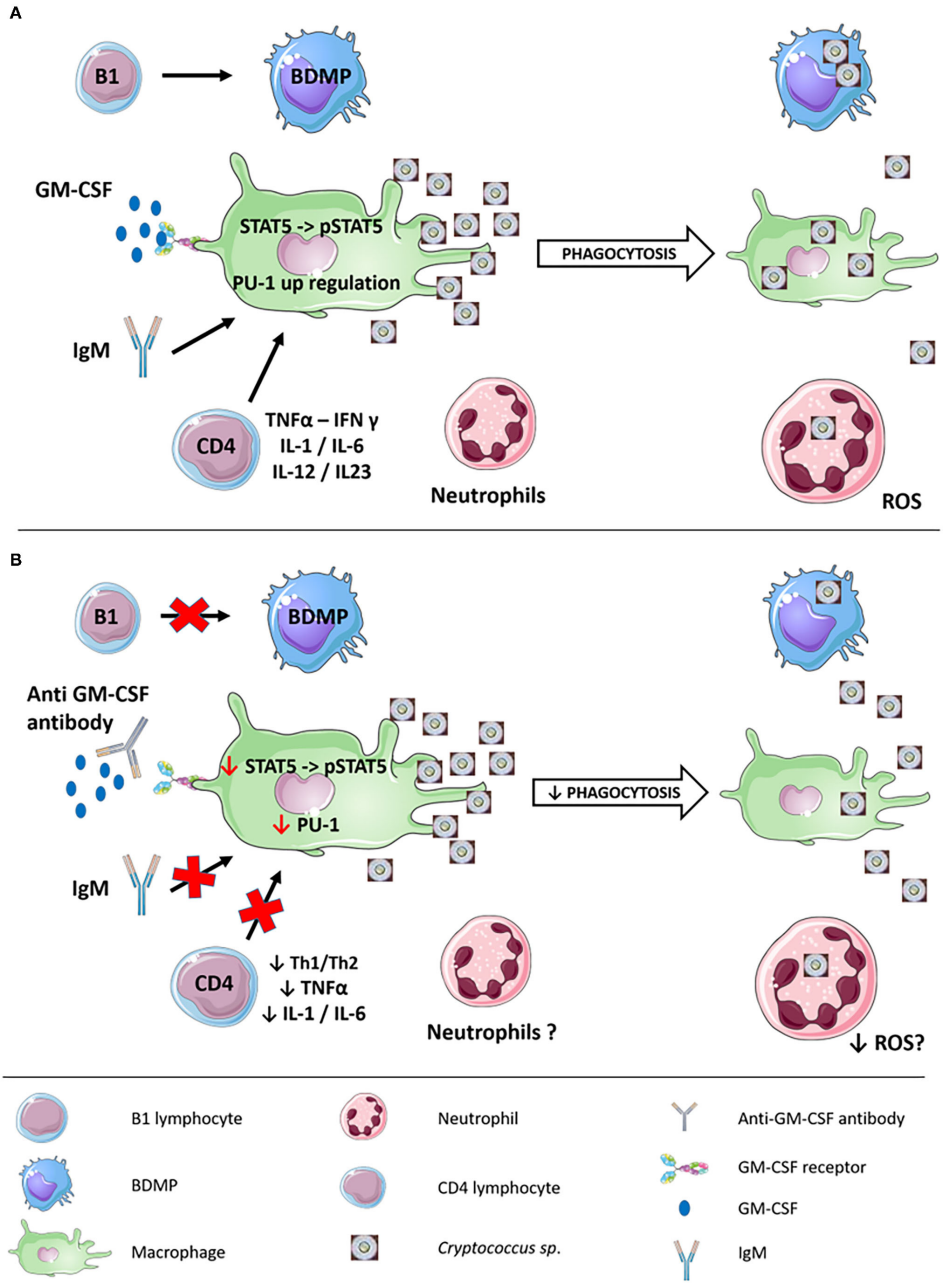
Immune response to cryptococcal infection and potential pathophysiological mechanisms responsible for the association between sarcoidosis and cryptococcosis. **(A)** Immune response to cryptococcal infection involving both innate and adaptative immunity. B1 cells can differentiate into BDMP with phagocytic activity against *Cryptococcus* spp. GM-CSF, IgM, and CD4 activate macrophage phagocytic activity, partly *via* the phosphorylation of STAT-5 upregulating the transcription factor PU-1. Neutrophils exert a fungicidal activity through the production of ROS. **(B)** Potential pathophysiological mechanisms responsible for the association between sarcoidosis and cryptococcosis. B1 cells could have impaired differentiation leading to defective BDMP phagocytic activity. Lack of IgM and CD-4 qualitative defects could lead to a defect in macrophage phagocytic activity. The presence of anti-GM-CSF antibodies could also prevent STAT-5 phosphorylation downregulating the transcription factor PU-1. Whether ROS species neutrophils production is impaired in sarcoidosis remains unknown. B-1, B-1 lymphocytes; BDMP, B-1-derived mononuclear phagocytes; CD4, CD4 lymphocytes; GM-CSF, granulocyte-macrophage colony-stimulating factor; ROS, reactive oxygen species.

Cryptococcosis is known to develop in patients with AIDS and idiopathic CD4 lymphopenia underlining the importance of CD4 T cells in the defense against *Cryptococcus* spp. (37). Systemic CD4 cells anergy is a feature of sarcoidosis as lymphopenia is correlated with disease severity during sarcoidosis (38) due to the accumulation of CD4 T lymphocytes in active granulomas participating in the “immune paradox” described in sarcoidosis: despite an extensive local inflammation, systemic anergy may develop (39). Nevertheless, in the cryptOsarc study, CD4 lymphocytopenia was not an independent risk factor for cryptococcosis (34) consistent with our findings that in CINS patients with sarcoidosis had a higher CD4 T cell proportion and CD4/CD8 T cells ratio than non-sarcoidosis patients. The authors of the cryptOsarc study even considered that “the CD4 levels in this study did not explain cryptococcosis in sarcoidosis”. If quantitative alteration of CD4 T cells function is not responsible *per se* for this association with cryptococcosis, alteration of qualitative CD4 T cell function could be involved in the pathophysiology (Figure 1), but T cell dysfunction in sarcoidosis is poorly understood so far. Data on peripheral CD25^high^ regulatory T cells (Treg) are contradictory; some authors reported a peripheral expansion that contributed to anergy, whereas others have reported a decrease in Treg cells with an imbalance in favor of Th17 cells (3).

Besides this potential qualitative CD4 T cell deficiency, an altered CD4 T cells-macrophage crosstalk could also be involved *via* decreased macrophage ability to contain *Cryptococcus* spp. (40). This hypothesis is reinforced as patients with X-linked CD40L deficiency or interleukin 12 receptor mutations exhibit higher susceptibility for cryptococcal infections (37). CD40L^−/−^ mice exhibited exacerbation of infection with a high fungal burden due to diminished interferon-γ production by CD4 and CD8 cells and decreased CD28 expression by CD4 cells (41). Moreover, nitrite production and antimicrobial activity by macrophages were impaired as was IL-12 production by splenic macrophages. Patients with auto-antibodies to interferon-γ and granulocyte-macrophage colony-stimulating factor (GM-CSF) also exhibit higher susceptibility to cryptococcal infections (37) as discussed below. The fact that interferon-γ and GM-CSF are known to be produced by CD4+ helper T cells to activate macrophages reinforce the plausibility of an altered CD4 T cells-macrophage crosstalk as a potential mechanism of increased susceptibility to cryptococcosis in sarcoidosis patients (42) (Figure 1).

Interest in the role of humoral immunity against *Cryptococcus* spp. was regained after the recent report of an association between invasive fungal infections, including cryptococcosis (43–45), and ibrutinib, an irreversible inhibitor of Bruton's tyrosine kinase (BTK). This clinical finding is strengthened by the fact that X-linked immunodeficient mice, which possess a mutation leading to a defective BTK, lack B-1 cells and natural IgM. These mice exhibit very high fungal burden after challenge with *C. neoformans* and fungal dissemination to the brain (46) consistent with previous findings in C57BL/6 mice depletion of B-1 cells (47) or sIgM^−/−^ (48). Conclusions from these studies were flawed by the presence of T cells in those models and by associated defects in cellular immunity (XID mice) and in B cell development (sIgM^−/−^ mice). To overcome these limitations, Rag1-deficient mice, which lack both T and B cells, were used, and B cells purified from wild-type animals were transferred (49) restoring B-1a and B-1b cells but not T cells or B-2 cells (50). This transfer led to a marked reduction in the cryptococcal burden in the brain but not in the lungs of the mice. Interestingly, transfer of IgG-depleted, IgM-containing serum in Rag1-deficient mice increased in alveolar macrophage recruitment and phagocytic index suggesting that the host benefit could be mediated by IgM-induced macrophage activation rather than a direct interaction (48). Another hypothesis is that B-1 cells could migrate to the lungs and differentiate into macrophage-like cells called B-1-derived mononuclear phagocytes (BDMP) (Figure 1) even if mechanisms by which B-1 B cells traffic to *C. neoformans*-infected lungs remains unknown (47). Those BDMP cells have been demonstrated to phagocyte *C. neoformans via* a complement receptor 3-mediated pathway with a higher fungicidal activity than a macrophage (51).

Even if sarcoidosis and granuloma formation in sarcoidosis are normally considered T cell-mediated peripheral B cells seem to be anergic in patients with sarcoidosis (52). This observed anergy could be due to the decreased levels of NF-κB/p65 (53) or to the lack of co-stimulation from CD4+ helper T cells (54). Nevertheless, whether sarcoidosis is associated with an impaired IgM production or with deficient B-1 cells is still not known.

The real impact of B cells on susceptibility for cryptococcosis has also been questioned as invasive fungal infections are very rare in patients with X-linked agammaglobulinemia with BTK deficiency, probably because of residual BTK activity in myeloid cells (37). In fact, BTK is expressed in all bone marrow cell lineages except T cells and plasma cells (55). The increased susceptibility of X-linked immunodeficient mice seems to be mostly due to the inability of macrophages to phagocyte *Cryptococcus* spp. rather than impairment of humoral immunity (46).

Besides adaptive immunity deficiency previously discussed, innate immunity deficiency seems to be a key player in the susceptibility to *Cryptococcus* spp. infection. This deficiency of innate immunity could be indirectly due to the impaired crosstalk with adaptive immunity, i.e., impaired activation of macrophages by deficient CD4 T cells or by lack of IgM as previously discussed, but it can also be directly due to the intrinsic deficiency of innate immunity (Figure 1).

For instance, anti-GM-CSF antibodies have been isolated from the serum of apparently immunocompetent patients with cryptococcosis with or without pulmonary alveolar proteinosis (56–59). The addition of sera from the patients containing anti-GM-CSF antibodies impaired myeloid cells activation from controls in the presence of *C. gatii* (56). These antibodies are exclusively IgG and mostly IgG1 and are biologically active by inhibition of GM-CSF-induced macrophage inflammatory protein-1α expression (MIP-1 α) and signal transducer and activator of transcription-5 (STAT-5) phosphorylation in control peripheral blood mononuclear cells (58–60). This inhibition reduces myeloid cells proliferation and differentiation but also their phagocytic and bactericidal capacities *via* the inhibition of PU.1 transcription factor (59, 61) (Figure 1). Anti-GM-CSF antibodies are also involved in pulmonary alveolar proteinosis (PAP), but most of the cryptococcosis patients presenting with anti-GM-CSF antibodies did not suffer from PAP (62). This might be explained by the extreme heterogeneity of those antibodies regarding their avidity and the targeted GM-CSF epitopes (63). Moreover, multiple clones of anti-GM-CSF antibodies could be present in the same patient.

Anti-GM-CSF antibodies could be part of an explanation for this association between cryptococcosis and sarcoidosis as a potential association has been described between PAP and sarcoidosis (64, 65). A recent study found in 5/92 (5.4%) patients with sarcoidosis to have detectable anti-GM-CSF antibodies, two of them with clinical PAP (62). Those patients exhibited significantly higher serum levels of Krebs von den Lungen-6, surfactant protein-D, lactate dehydrogenase and required more often systemic corticosteroid therapy. It would thus be interesting to assess if anti-GM-CSF antibodies are more prevalent in patients with sarcoidosis and suspicion of cryptococcal infection. If so, the presence of those antibodies could help to identify at-risk patients. Nevertheless, whether uptake by macrophages is a conclusive readout for protecting against cryptococcal invasion is still unclear. In fact, recruited M1 polarized monocyte-derived macrophages are thought to have fungicidal activity contrary to alveolar macrophages (66). Alveolar macrophages could even be involved in the cryptococcal dissemination outside the lungs (67, 68).

Another major effector of first-line defense against cryptococcal infection is the pool of neutrophils as they have been demonstrated to engulf and kill *Cryptococcus* spp. more efficiently than monocytes (69) and produce reactive oxygen species (ROS) that kill *Cryptococcus* spp. (70). However, the role of neutrophils in cryptococcal infections is not straightforward as, in contrast to intravascular infection, they seem to worsen the prognosis in the setting of intratracheal infection (71, 72). Absolute neutrophil count of more than 3,500 cells/mm^3^ is even associated with increased mortality in HIV-infected patients with cryptococcal meningitis (73). This could be explained by the fact that neutrophils are recruited to the lungs in response to cryptococcal infection by lung parenchymal lymphocytes and that T cells (CD4 cells but also CD8 cells and γδ cells) impairment is associated with a compensatory neutrophil response requiring IL-17A, which worsens lung injury (71, 72, 74). Moreover, the following two distinct neutrophil subsets seem to be generated in response to cryptococcal infection: (i) one with an oxidative stress signature interacting directly with the fungus and generating ROS and (ii) another with enhanced cytokine gene expression which are longer-lived and that indirectly respond to cryptococcal ligands to modulate crosstalk, *via* the expression of IL-1α, TNFα, and complement C3, with dendritic cells and alveolar macrophages through CCR5 and CCR1, respectively (75). Mechanisms responsible for such a differentiation remain unknown. While in contact, *Cryptococcus* spp. phagocytosis by neutrophils is dependent on the complement C5a-C5aR axis (76).

Very less is known about neutrophil's involvement in sarcoidosis pathophysiology except for that neutrophil/lymphocyte ratio in the complete blood count can be used as an indicator of inflammation in sarcoidosis (77). Although neutrophil accumulation-inducing chemokines like IL-8 are increased in sarcoidosis, the percentage of neutrophils in the bronchoalveolar lavage remains low and is not correlated to CXCL8 or CXCL5 as in idiopathic pulmonary fibrosis (78–80). Nevertheless, the percentage of neutrophils in bronchoalveolar lavage is associated with progressing sarcoidosis and an increased risk for corticosteroid therapy (81, 82). The role of neutrophils in the association between sarcoidosis and cryptococcosis thus remains to be demonstrated (Figure 1).

## Conclusion

Sarcoidosis was the most frequently associated condition with CINS in this study. CINS patients with associated sarcoidosis had overall similar clinical and biological presentation than CINS patients associated with other conditions but exhibited a lower rate of positive blood cryptococcal antigen testing and higher CD4/CD8 T cells ratio. Pathophysiological mechanisms underlying this association remain poorly understood, but B-1 cell deficiency or lack of IgM could be a part of the explanation. Another plausible mechanism is the presence of anti-GM-CSF antibodies in a subset of patients with sarcoidosis that could impair macrophage phagocytic function. Further studies are strongly needed to better understand those mechanisms and to identify at-risk patients.

## Data Availability Statement

The raw data supporting the conclusions of this article will be made available by the authors, without undue reservation.

## Ethics Statement

Ethical review and approval was not required for the study on human participants in accordance with the local legislation and institutional requirements. Written informed consent for participation was not required for this study in accordance with the national legislation and the institutional requirements.

## Author Contributions

RP, LD, PB, and PD contributed to the conception and design of the study. RP, SI, and VG contributed to the data collection. RP and PD wrote the manuscript. Each author drafted or provided critical revision of the article and provided final approval of the version submitted for publication.

## Conflict of Interest

The authors declare that the research was conducted in the absence of any commercial or financial relationships that could be construed as a potential conflict of interest.

## Publisher's Note

All claims expressed in this article are solely those of the authors and do not necessarily represent those of their affiliated organizations, or those of the publisher, the editors and the reviewers. Any product that may be evaluated in this article, or claim that may be made by its manufacturer, is not guaranteed or endorsed by the publisher.
